# The Effect of Long-Term Changes in Daily Stress Processes on Prospective Health: An Application of Three-Level SEM

**DOI:** 10.1093/geroni/igaa057.1645

**Published:** 2020-12-16

**Authors:** Jonathan Rush, Emily Willroth, Eileen Graham, Daniel Mroczek, David Almeida

**Affiliations:** 1 University of Victoria, Victoria, British Columbia, Canada; 2 Northwestern University Feinberg School of Medicine, Evanston, Illinois, United States; 3 Northwestern University, Chicago, Illinois, United States; 4 Pennsylvania State University, University Park, Pennsylvania, United States

## Abstract

The study of change over time, contexts, cohorts, and people is influenced by the sampling of observations within longitudinal studies. Intensive measurement designs, embedded within long-term longitudinal studies, provide new opportunities to understand changes in dynamic processes, as well as determinants and consequences of these changes over time. The present investigation examined whether short-term dynamic associations accounted for individual differences in prospective health functioning. We used measurement burst data from the National Study of Daily Experiences subsample (N = 2485) embedded within the Midlife in the United States longitudinal study. Two measurement bursts were separated by ten years, with each containing daily measures of stress and affect across eight consecutive days. Functional health was measured by basic and instrumental activities of daily living at three measurement waves spanning 20 years. Three-level structural equation models were fit to simultaneously model short-term within-person associations between stress and affect (i.e., stress reactivity) and long-term changes in these associations over the ten year period. Individual differences in long-term changes of the short-term dynamic association predicted both basic and instrumental activities of daily living at 20 year follow-up (estimate = 5.26, SE = 2.54, p < .01; and estimate = 5.48, SE = 2.81, p < .01, respectively). These effects were present after adjusting for mean levels of both stress and affect. We highlight how characterizing individuals based on the strength of their within-person associations across multiple time scales can be informative in predicting distal health outcomes.

